# IDICAP: A Novel Tool for Integrating Drug Intervention Based on Cancer Panel

**DOI:** 10.3390/jpm6040019

**Published:** 2016-10-28

**Authors:** Noelle Kosarek, Eric S. Ho

**Affiliations:** 1 Department of Biology, Lafayette College, Easton, PA 18042, USA; 2 Current address: Geisel School of Medicine at Dartmouth College, Hanover, NH 03755, USA; Noelle.N.Kosarek@dartmouth.edu

**Keywords:** cancer gene panel, precision medicine, targeted cancer therapy, clinical trials, DrugBank, protein-protein interactions, DGIdb, OpenGeneMed

## Abstract

Cancer is a heterogeneous disease afflicting millions of people of all ages and their families worldwide. Tremendous resources have been and continue to be devoted to the development of cancer treatments that target the unique mutation profiles of patients, namely targeted cancer therapy. However, the sheer volume of drugs coupled with cancer heterogeneity becomes a challenge for physicians to prescribe effective therapies targeting patients’ unique genetic mutations. Developing a web service that allows clinicians as well as patients to identify effective drug therapies, both approved and experimental, would be helpful for both parties. We have developed an innovative web service, IDICAP, which stands for Integrated Drug Intervention for CAncer Panel. It uses genes that have been linked to a cancer type to search for drug and clinical trial information from ClinicalTrials.gov and DrugBank. IDICAP selects and integrates information pertaining to clinical trials, disease conditions, drugs under trial, locations of trials, drugs that are known to target the queried gene, and any known single nucleotide polymorphism (SNP) effects. We tested IDICAP by gene panels that contribute to breast cancer, ovarian cancer, and cancer in general. Clinical trials and drugs listed by our tool showed improved precision compared to the results from ClinicalTrials.gov and Drug Gene Interaction Database (DGIdb). Furthermore, IDICAP provides patients and doctors with a list of clinical facilities in their proximity, a characteristic that lends credence to the Precision Medicine Initiative launched by the White House in the United States in 2015.

**URL:** http://idicap.lafayette.edu:8000

## 1. Introduction

The causes of cancer are vast and range from genetic alterations and environmental factors to infection and hormonal influence. Additionally, recent studies found that intra-tumor and inter-tumor variations are prevalent in breast cancer, lung cancer, and medulloblastoma [1,2,3,4]. Cancer heterogeneity posts great challenges for diagnosis and treatment. Chemotherapy has remained as one of the most widely utilized cancer treatments in medical practice. Chemotherapeutic drugs, such as paclitaxel, work by disrupting the assembly of microtubules, hampering cell division, and triggering apoptosis in cancerous as well as healthy cells [5]. Its non-specificity causes undesirable side effects in patients. These include nausea, vomiting, pain especially in the feet and hands, and blood disorders, among others [6]. To overcome the adverse effects produced by chemotherapy, researchers are developing therapies that specifically target cancer-driving genes, aiming at minimizing off-target effects and maximizing efficacy. This strategy is termed targeted therapy. There has been varying degree of success depending on the mechanism of action and the type of cancer. Gleevec offers a much more direct intervention for reversing chronic myeloid leukemia, achieving an 89% survival rate in 60 months [7]. Herceptin targets the HER2 gene in early breast cancer patients demonstrating a 24% survival rate [8]. Both treatments provide better tolerated options while delivering survival benefits. These two success stories, therefore, lead to the burgeoning of targeted cancer therapy. Drugs currently labeled with gene targets were found in 22% (1815 out of 8206) of the drugs in DrugBank [9] according to our analysis. Out of the 1815 drugs, 341 were labeled as cancer drugs (see Materials and Methods for details). With the launch of the Precision Medicine Initiative (PMI) by the White House in 2015 [10], we anticipate the number of targeted drug therapeutics to rise, inundating clinicians and patients with a fount of treatment information.

**Figure 1:**
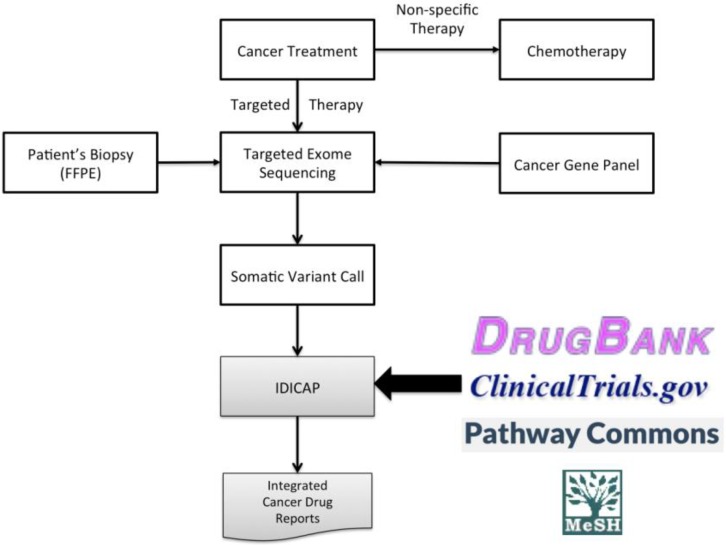
A roadmap of cancer therapy: non-specific and targeted therapies. IDICAP (Integrated Drug Intervention for CAncer Panel) plays the role in integrating drug and clinical trial information that treat the unique mutation profile of patients. FFPE: formalin-fixed, paraffin-embedded.

Figure 1 highlights targeted therapies among other cancer treatments and the role of our tool IDICAP, an acronym for Integrated Drug Intervention for CAncer Panel, in the proposed treatment roadmap. An effective therapy begins with an accurate portrayal of the patient’s gene mutation profile. The advance of DNA short-read sequencing technology, better known as Next Generation Sequencing (NGS), provides a powerful technology to capture the unique mutation landscape of patients. It is known that mutations of a handful of genes, namely the cancer panel genes, drive cancer development, although cancer panel genes of some cancers are better understood than the others. Additionally, a majority of deleterious somatic mutations occur in exons, resulting in aberrant proteins or complete gene knockout. Thus, channeling sequencing capacity of NGS to well-supported pathological exons and regulatory regions of cancer panel genes is a more effective approach to harness the power of NGS than sequencing all genes or the whole genome. The benefit includes higher sequencing depth and quality but at a reduced cost per base. Besides, physicians can use certain functional regions as markers to monitor the cancer progression of their patients [11]. This sequencing approach is known as targeted exome sequencing. A study in 2013 [12] that sought to test the feasibility of a targeted sequencing platform for explorations of colorectal cancer utilized a set of 183 genes that directed their project. The researchers determined this set of genes was sufficient for indicating which genes were most often mutated or regulated inappropriately in colorectal cancer.

The genetic testing industry has grown rapidly in recent years. Many diagnostics companies use NGS or microarrays to identify genetic variations through cancer gene panels [12]. The genetic testing company Color Genetics [13] has created a cancer panel for breast and ovarian cancers that include the genes *ATM*, *BARD1*, *BRCA1*, *BRCA2*, *BRIP1*, *CDH1*, *CHEK2*, *EPCAM*, *MLH1*, *MSH2*, *MSH6*, *NBN*, *PALB2*, *PMS2*, *PTEN*, *RAD51C*, *RAD51D*, *STK11*, and *TP53*. Doctors can perform a biopsy of a patient’s tumor and send the formalin-fixed, paraffin-embedded (FFPE) tissue sample to a diagnostic company for targeted exome sequencing. Following exome sequencing is the detection of genetic variations: point mutation, short indel, copy number variation, gene duplication, and gene fusion, through statistical and bioinformatic analyses.

Producing a genetic variation report is usually the final step in the current diagnosis practice, leaving physicians to search for treatments that address the particular genetic variations affecting the patients. As mentioned above, there are 341 cancer drugs that are designed to target a myriad of genes, and even more are undergoing clinical trials. At the time of writing, almost 8000 cancer drug clinical trials are undergoing different phases of trials according to the ClinicalTrials.gov website [14]. For breast cancer alone, 477 clinical trials are recruiting patients across the United States. Navigating through such a deluge of ever-changing information is time-consuming and burdensome for physicians and patients, not to mention the stress and looming financial cost that plagues patients. The goal of our IDICAP tool is to advance the targeted therapy field by integrating up-to-date targeted therapeutic information for physicians based on the unique mutation profile of cancer patients when the current standard of care has been exhausted or is inadequate (Figure 1). IDICAP reports contain cancer drugs aimed at gene targets including those that are already on the market or still undergoing clinical trials. Taking part in clinical trials offers a viable alternative treatment option when the standard of care is suboptimal. The benefits include the access to treatment that is currently not available in standard clinical environments, the access to advanced facilities staffed by highly trained personnel as clinical trials usually take place in well-equipped medical centers such as university hospitals, and subsidies for treatment cost. Furthermore, clinical trials have the potential to advance medicine such that others who have suffered from the same ailment can routinely be treated in the future if the investigational drug is proven efficacious and safe. We compared our results to the output produced by Drug Gene Interaction Database (DGIdb), a web service that serves similar functions [15]. Although it is hard to make a simple comparison between the two as each has its own unique features, our results tend to be more specific toward drugs designed for targeting particular genes.

## 2. Results

### 2.1. Breast Cancer Panel

**Figure 2:**
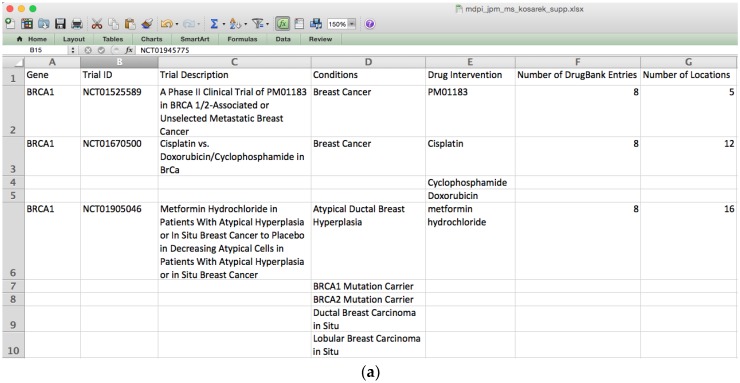

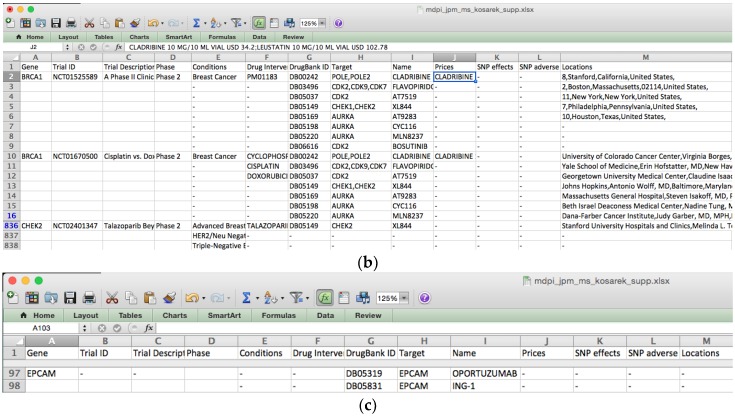
IDICAP reports. In each execution, IDICAP produces two tab-delimited reports that can be viewed conveniently by common spreadsheet programs such as Microsoft Excel, Google Sheets, or OpenOffice Calc. (**a**) Summary report matches open clinical trials and drug information, if any, per gene. It consists of seven columns: queried gene symbol, unique reference number of the clinical trial assigned by ClinicalTrials.gov, title of the clinical trial, disease conditions studied by the trial, names of drugs under trial, number of drugs targeting the queried gene discovered in DrugBank [17], and number of locations conducting the current clinical trial. (**b**) Detailed report consists of 13 columns. Columns A-C, E, and F are inherited from the summary report. Phase of the clinical trial (column D) is relevant to assess the stage of the clinical trial. Columns G through L are information obtained from DrugBank regarding the queried gene. Column H indicates the gene(s) targeted by the drug (columns G and I) as documented in DrugBank. A gene in this column is either the queried gene (column A) or the gene(s) interacting with the queried gene (see Section 2.2 for details). Column I is the drug name in DrugBank. Column J is the reference drug price for various dosages, if any. Columns K and L are known single nucleotide polymorphism (SNP) effects of the drug, if any. The last column contains contact information e.g., address, contact person, etc., of the clinical trial facilities. Note that the DrugBank information in columns G-L is repeated for different clinical trials of the same gene so that users can simply read a single report to obtain all relevant information about clinical trials and drugs. (**c**) No clinical trial is found to associate with the gene *EPCAM*. Only drug information is listed in the report.

A set of 11 genes were obtained from the website of the genetic testing company Color Genomics, Inc. Color Genomics uses a single cancer panel to assess an individual’s hereditary risk of breast cancer. These 11 genes were used to demonstrate the features of IDICAP (Supplementary File Table S1). The selection of these genes is supported by studies in which clinical evidences support their association with the onset of breast cancer. In each run, IDICAP produces two reports with different levels of detail, namely a summary report and a detailed report. Figure 2 below shows a snapshot of the two reports. Full reports for various cancer gene panels tested in this study can be found in Supplementary File Tables S5-10.

In this example, IDICAP sent 11 queries, one for each gene, to ClinicalTrials.gov in real time sequentially. After filtering (discussed below) the returned results, 20 clinical trials and 32 drugs were identified to be associated with 10 out of the 11 genes in the breast cancer panel. Note that ClinicalTrials.gov does not index clinical trials by gene symbols therefore the search for clinical trials by genes is handled as a keyword search by the website. It means that further screening of the returned results is mandatory in order to discard spurious clinical studies. IDICAP uses the following criteria to eliminate irrelevant trials. Under our screening scheme, a trial must:

1. Mention the queried gene symbol: returned clinical trials must contain the queried gene symbol but are not restricted to any fields in the clinical trial record. This criterion does not guarantee that the returned clinical trials are designed specifically to target the queried gene. For example, a non-cancer trial NCT02246491 (Cell-Based Approaches For Modeling and Treating Ataxia-Telangiectasia) was returned even when we searched for cancer trials related to the gene *ATM*. Thus, additional filtering criteria (see below) are needed to effectively remove specious trials.
2. Test a cancer treatment: For example, though breast neoplasm was specified in the condition field of a query that is sent to ClinicalTrials.gov, not all returned clinical trials were actually breast cancer trials. For instance, trial NCT01591746, a breast reconstruction surgery trial, was returned from the query. To circumvent specious trials, we pre-compiled a list of neoplasm-related Medical Subject Headings (MeSH) acquired from the U.S. National Library of Medicine [16]. IDICAP requires a trial to contain at least one neoplasm-related MeSH term in the disease condition subsection.
3. Involve a drug intervention: our tool focuses on cancer drug trials. However, non-drug trials were returned occasionally even though drug intervention was specified in the query. For example, a dietary supplement trial (NCT01975363) for women with obesity and high risk for breast cancer was returned. To filter out non-drug trials, our tool enforces that a drug compound name must be listed under the intervention subsection.
4. Recruit patients actively: a trial must be currently recruiting patients and it must be conducted in the country or countries specified by the user, if any. Users in the United States have an extra option to limit trial facilities in the vicinity of a zip code. For instance, the first trial (NCT01525589) in Figure 2a,b is conducted in five facilities spread across the United States: California, Massachusetts, New York, Pennsylvania, and Texas. In normal cases, patients residing on the East Coast would not travel to the West Coast to participate in a trial that usually entails multiple visits to that distant site. IDICAP allows users to restrict the maximum distance of facilities from a zip code. For example, IDICAP can list treatment sites within 50 miles from the zip code 18042 (Easton, Pennsylvania). In this case, the number of facilities would be reduced to two, i.e., New York and Pennsylvania.

It is noteworthy that some clinical trials are designed to target multiple genes. For example, trial NCT02401347 evaluates the anti-cancer activity of the drug talazoparib in patients with advanced breast cancer. The trial is designed to recruit two cohorts. One of the cohorts consists of HER2-negative breast cancer patients with somatic mutations in one of the following genes: *PTEN*, *PALB2*, *CHEK2*, *ATM*, *NBN*, *BARD1*, *BRIP1*, *RAD50*, *RAD51C*, *RAD51D*, *MRE11*, and *ATR*. As a result, this trial was reported multiple times by IDICAP, once for each queried gene symbol.

### 2.2. Incorporation of Drug Information from DrugBank

To provide additional therapeutic options for physicians and patients, IDICAP not only extracts clinical trial information, but also integrates drug information from a comprehensive drug database, DrugBank [17]. DrugBank stores information for both on-the-market and investigational drugs. The queried gene symbol is used as the key to search through the targeted gene field of each drug annotated in the DrugBank database. Figure 2b (row 836) illustrates a trial (NCT02401347) for the drug talazoparib which targets the gene *CHEK2* for triple negative breast cancer patients. In conjunction with DrugBank, the cancer drug XL844 (DB05149) is also identified to inhibit *CHEK2*.

On the other hand, not every gene in the cancer panel is associated with clinical trials. For instance, no trial has been identified to target *EPCAM* for ovarian cancer patients (Figure 2c). But IDICAP is still able to identify two *EPCAM*-target drugs, oportuzumab monatox (DB05319) and ING-1 (DB05831) from DrugBank, demonstrating the added value of IDICAP.

**Figure 3:**
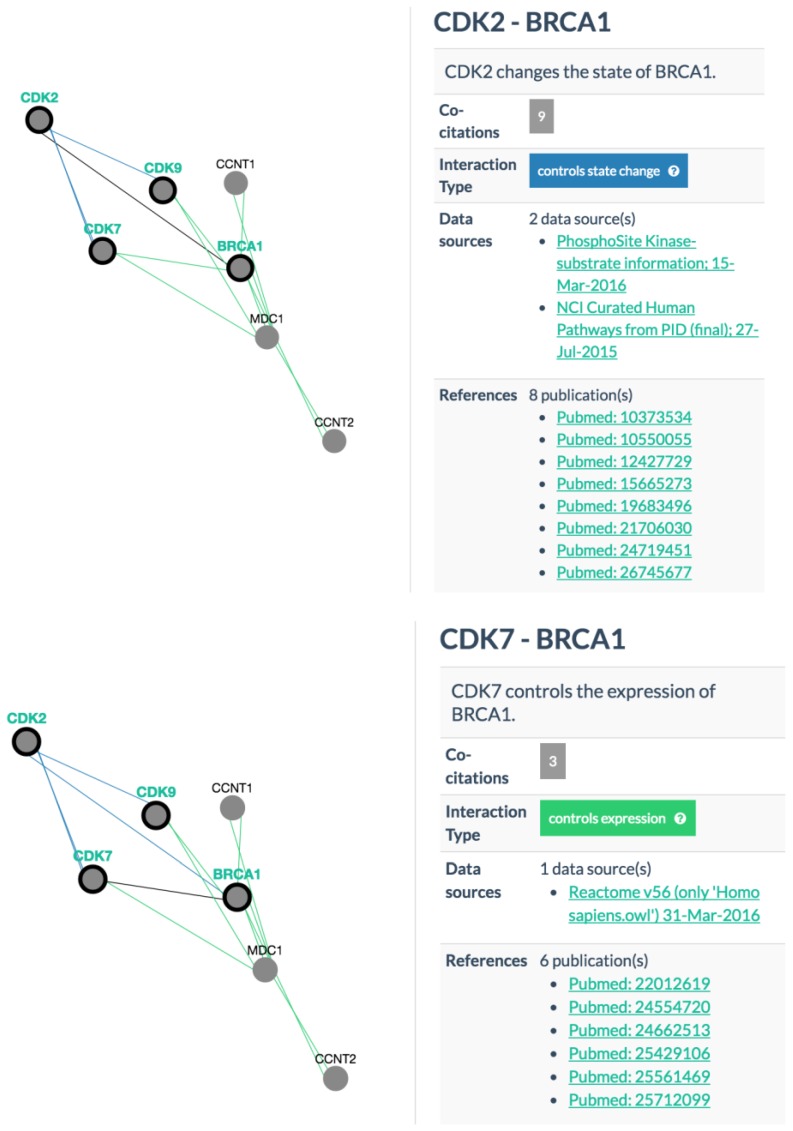

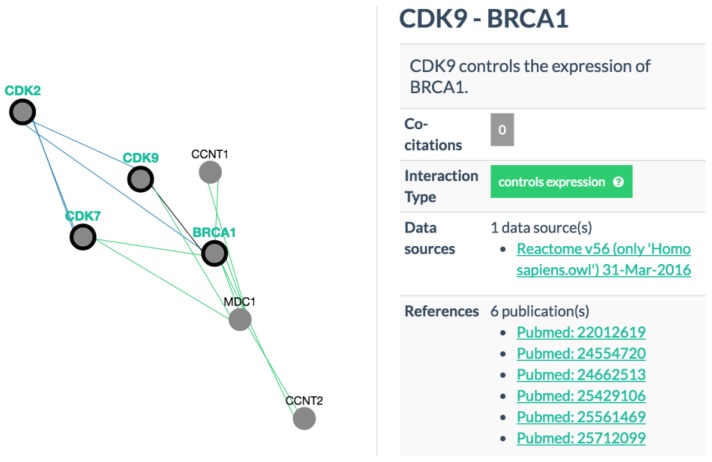
Protein-protein interactions. Screenshots taken from PCViz (Pathway Commons Network Visualizer) for interactions CDK2-BRCA1, CDK7-BRCA1, and CDK9-BRCA1 in which CDK2 controls the state of BRCA1 and CDK7/9 control the expression of BRCA1. We assume that interfering with the function of CDK2/7/9 may affect BRCA1 as well. CDK: cyclin-dependent kinase; BRCA1: breast cancer 1.

It should be noted that many cancer genes are not directly targeted by drugs. We are inspired by the story of repurposing the kidney cancer drug sunitinib or sutent (DB01268) for treating acute lymphoblastic leukemia (ALL) and acute myeloid leukemia (AML) patients[18,19,20] by antagonizing the elevated activity of FMS-like tyrosine kinase-3 (FLT3) present in a rare form of ALL patients. When no drug is found by IDICAP in DrugBank for the queried gene, IDICAP uses protein-protein interaction (PPI) data downloaded from Pathway Commons [21] to infer genes that may mediate the activity of the queried gene. As most drugs are antagonists, drugs that target the regulators or the partnering proteins of a complex of the queried gene may also antagonize the activity of the queried gene. For the example in Figure 2b, the query for gene *BRCA1* (row 2) yielded no results from DrugBank. With the inclusion of PPI data, IDICAP identified genes *CDK2*, *CDK7*, and *CDK9* (column H in Figure 2b) that control the state or the expression of *BRCA1* (Figure 3).

Our tool used CDK2, CDK7, and CDK9 to repeat the search for drugs that target BRCA1’s regulators in DrugBank. It found that the drug flavopiridol (DB03496 in column I row 3 of Figure 2b), which treats a number of cancers including esophageal, lung, liver, and lymphoid leukemia, could be used to treat patients with mutations in BRCA1. Although the efficacy of repurposing flavopiridol for treating breast cancer demands further confirmative studies, IDICAP contributes to aid physicians in navigating through the maze of clinical data and identifying potential therapeutics already on the market for the treatment of other diseases, hoping that the success of repurposing sunitinib can be repeated.

### 2.3. Drug Reference Prices

Drug reference price in USD is listed in column J in the detailed report (Figure 2b). This information is extracted from DrugBank in which the price is based on the markets in the United States and Canada [22]. The actual cost of the drugs depends heavily on the patient’s country of residence as well as his or her health insurance plan. The rationale for including the drug price is to facilitate drug cost comparison and estimation. For example, the drug gefitinib (DB00317), which targets the gene *EGFR*, goes by multiple trade names and dosages in the market: Tarceva (by Genetech, San Francisco, CA, USA) 25 mg tablet USD 52.78, Iressa (by AstraZeneca, London, UK) 250 mg tablet USD 68.08, Tarceva 100 mg tablet USD 144.98, and Tarceva 150 mg tablet USD 163.98. As you can see from this example, drug price varies by brand name and dosage. The benefit of IDICAP is to aggregate scattered price information into a single report that assists physicians and patients in estimating the treatment cost under different options.

### 2.4. Single nucleotide polymorphism (SNP) Effect

Genetic variations influence absorption, distribution, metabolism, and elimination (ADME) of drug compounds. With the ever-decreasing DNA sequencing cost, personal genomics information becomes readily available, accelerating the discovery of pharmacogenomic biomarkers associated with drugs. The number of drugs that target SNPs has expanded by 10% and the number of SNPs known to cause potential negative side effects by drugs has grown by 78% [9,22]. For example, the drug gefitinib (DB00317) is sensitive to the G719A/C variation in the *EGFR* gene of patients suffering from non-small cell lung cancer. The rationale for including SNP effect and adverse events information in the detailed report (columns K and L Figure 2b) is to raise the awareness of the impact of genomic variations on ADME, resulting in personalized medication for optimal use.

IDICAP extracts SNP information from two sections of the DrugBank database: “snp-effects”, and “snp-adverse-drug-reactions”. In both sections, we are interested in extracting the protein name, gene name, SNP accession number, allele, and description per SNP.

### 2.5. Location of Clinical Facility

Providing patients with the exact locations of trials is integral to determining an optimal treatment. For example, a patient residing in the United States may not find it very useful to know that a trial related to his cancer is being performed in Japan. IDICAP provides the option for users to restrict potential trials to a particular country or a list of countries, e.g., the United States and Canada. If the United States is the country specified, our tool can narrow down the clinical facilities within a smaller region by the distance from a zip code, e.g., 50 miles from the zip code 18042. If the zip code information of a facility in the United States is unknown, the report simply includes it without filtering.

### 2.6. Comparison with Advanced Search Results at ClinicalTrials.gov

One may argue that IDICAP does precisely what the website ClinicalTrials.gov has been providing in terms of selecting clinical trials that satisfy certain criteria specified by users. A comparison of search results produced by IDICAP and ClinicalTrials.gov indicated that the filtering criteria adopted by IDICAP are more effective than ClinicalTrials.gov thereby identifying more relevant trials. We addressed the result precision issue between IDICAP and ClincialTrials.gov through a set of queries. The testing queries were formulated to search for cancer drug trials that were actively recruiting patients suffered from a specific type of cancer and with certain genes being mutated in the United States. For example, we searched for cancer drug trials that were recruiting breast cancer patients with *BRCA1* mutation in the United States. We used the Advanced Search input form in ClinicalTrials.gov to perform the test in which fields *Recruiting* was ‘-- Recruiting’, *Study Results* was ‘All Studies’, and *Study Type* was ‘Interventional Studies’. Under the *Targeted Search* subsection, *Conditions* was ‘breast neoplasm’, and *Interventions* was ‘drug’. For the *Locations* subsection, *Country1* was ‘United States’. The rest of the fields were left with default values. Table 1 below summarizes the query results returned for breast and ovarian cancer gene panels.

It is interesting to note that three clinical trials (NCT02595905, NCT02162719, and NCT02155777) returned by ClinicalTrials.gov were, in fact, not recruiting patients even though the query parameters required that the results return only those trials in the ‘Recruiting’ state. In addition, trial NCT02162719 involves no actively recruiting locations within the United States, though the query specified that the trial be in the United States. As the documentation regarding the internal algorithm underpinning ClinicalTrials.gov’s query engine is unavailable, the cause of the discrepancies remains unclear.

The goal of trial NCT01591746, which is mentioned in Table 1, is to establish the efficacy and safety of Botulinum Toxin A (Botox) in alleviating pain for women undergoing unilateral or bilateral mastectomies. It is outside the scope of targeted drug therapy; thus, it should not be listed in returned results. The MeSH terms relevant to the disease condition for that trial focused on conditions such as postoperative pain, breast diseases, etc., other than neoplasm. This example confirms the effectiveness of the screening criteria discussed previously in Section 2.1.

Lastly, NCT02477202 is a medical device trial for patients suffering from ovarian cancer. No drug information is provided in the intervention subsection of the trial record. We are unsure of the reasons why it was returned by ClinicalTrials.gov even when the *Intervention* parameter was set to “drug”.

In summary, the comparison suggests that IDICAP’s screening criteria are effective in boosting precision of trials returned from ClinicalTrials.gov. In particular, the incorporation of MeSH terms suffices in enhancing the precision of our search results.

### 2.7. Comparison with DGIdb

Discovering drugs targeting queried genes performed by IDICAP is analogous to identifying interactions between drugs and cellular proteins or drug-gene interactions referred by some databases. Thus, we compared the queried results between IDICAP and a comprehensive drug-gene interaction database DGIdb [15,23]. The DGIdb database stores semi-automatic annotations of gene-drug interactions obtained from 15 sources. We used the comprehensive oncogene panel (Supplementary File Table S3) provided by Admera Health to compare the query results returned by IDICAP and DGIdb. In order to make the search results comparable, only DrugBank was selected in the list of source databases in DGIdb as IDICAP obtained drug information from DrugBank only. Table 2 below highlights only a small subset of genes for discussion. The full comparison of 56 genes can be found in Supplementary File Table S11: DGIdb vs. IDICAP.

The gene product of *ABL1* is a drug target for several drugs. IDICAP and DGIdb shared six drugs that are known to target *ABL1*. The three chemical names listed by DGIdb (DB08043, DB07831, and DB08350) were excluded by our tool because DrugBank does not annotate whether or not they are cancer drugs. They are probably still in the early stage of development. Adenosine triphosphate (DB00171), which was returned by DGIdb only, which targets 33 genes including *ABL1* according to DrugBank. However, its main usage is in nutritional supplementation rather than cancer treatment. IDICAP suggested XL228 but not DGIdb. XL228 is an experimental drug for treating Acute Lymphocytic Leukemia. Phase 1 study of the compound was completed in 2011 (NCT00464113). Obviously, the potency of the compound requires further clinical investigation. However, our goal is to inform physicians of the existence of such therapeutics, even though it is still under development, so that physicians may recommend that their patients be enrolled in the trial due to the absence of promising standard treatments.

The second gene highlighted in Table 2 is *ATM*. Caffeine (DB00201) was returned by DGIdb but it was excluded by IDICAP because it is not a cancer drug. Additionally, we did not find any cancer drugs aimed directly at ATM in DrugBank. Thus, IDICAP searched for genes that interact with ATM with the rationale discussed above (Section 2.2). Along this line of thought, our tool discovered that *ATM* interacts with *POLE*, *POLE2*, *CDK7*, *CDK9*, *CHEK1*, and *CHEK2* in which drugs are found to attenuate their activities. Cladribine (DB00242) targets *POLE* and *POLE2* genes and it is mainly used to treat chronic lymphocytic leukemia (CLL). *CDK7* and *CDK9* are targeted by flavopiridol (DB03496), which is a cancer drug treating various types of cancer.

BRCA1 (breast cancer 1) performs DNA repair functions and it is normally expressed in breast tissue. Mutations in *BRCA1* are associated with the onset of breast cancer. Unfortunately, no drugs are on the market or under trial that target *BRCA1* mutations in breast cancer patients. By making use of protein-protein interaction information, we suggested eight cancer drugs that target genes upstream of *BRCA1*.

Our results largely agree with DGIdb if the queried gene is a known target of drugs. However, what makes IDICAP unique is our focus on cancer therapeutics instead of general drug-gene interactions provided by DGIdb. It also meets our goal, which is to provide information for physicians when existing standards of care have been exhausted.

### 2.8. Comparison with OpenGeneMed

At the time of writing, a new tool, OpenGeneMed, has been published [24]. It is an open source tool that provides clinicians, researchers, and lab staff with a shared platform for coordinating the progress of patients enrolled in clinical trials. The tool integrates patient information, lab testing results, somatic mutation calls, gene/drugs associations, and treatment rules. Though OpenGeneMed adequately facilitates communication among professionals, its underlying design principles seem to target for sizeable facilities in the United States. We believe that OpenGeneMed can benefit patients who can access facilities that are staffed by a wide variety of health professionals mentioned above. However, OpenGeneMed does not offer open access web service, meaning that it is only available within certain organizations. In contrast, IDICAP offers clinicians or patients who affiliate with small private practices or users outside the US healthcare system the functionalities to explore therapeutic options advanced by precision medicine technology.

## 3. Discussion

The goal of this project was to develop a user-friendly web service that aids both physicians and patients in gathering, filtering and integrating clinical trial and drug information related to cancer genes, ultimately leading to the advancement of personalized treatment of cancer patients. As targeted therapy is gradually occupying the center stage in cancer treatment, both physicians and patients are facing difficulties in formulating effective treatment plans. Our web service pulls relevant and current clinical information effortlessly and promptly, making cancer therapeutic information freely accessible to a wide audience.

Although ClinicalTrials.gov also supports complex queries, our comparison suggests that IDICAP’s screening criteria are more effective in returning relevant trials, resulting in high precision, and saving physicians’ time in exploring treatment options for patients. When compared with DGIdb, the drugs listed by IDICAP are closely related to cancer treatment though they have not been thoroughly tested for the treatment of certain cancer types. Our tool provides a highly accessible service to patients and doctors in the niche of targeted therapy compared with OpenGeneMed. Furthermore, our tool explores a new avenue to repurpose existing therapeutics for other cancer types through protein-protein interaction information. The results show that a sizeable pool of drugs, either approved or experimental, target upstream mediators of a queried gene or its partnering proteins in a complex. This result is promising even though further thorough studies are needed to support the efficacy.

## 4. Materials and Methods

### 4.1. An Overview of IDICAP Web Service

**Figure 4:**
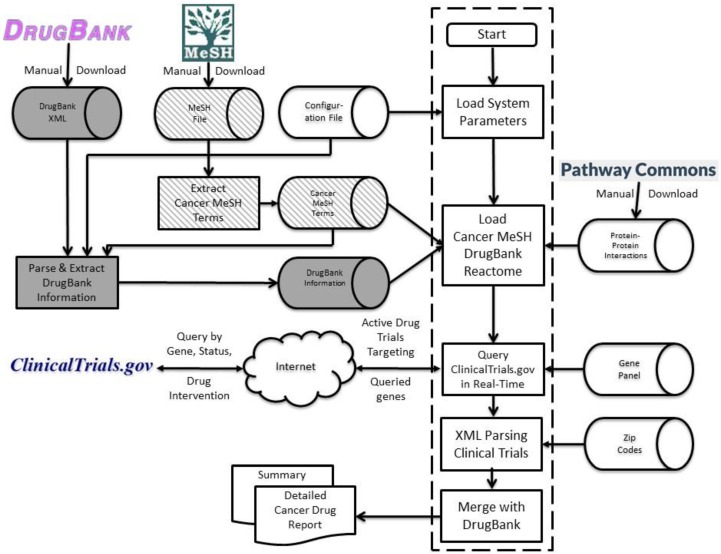
System architecture of IDICAP. The solid shaded components pertain to the process to parse and extract relevant drug information from DrugBank. The diagonally shaded components represent the process to select neoplasm Medical Subject Headings (MeSH) terms from the MeSH file downloaded from the National Library of Medicine. The components inside the dashed box form the IDICAP processing. IDICAP requires six files to run: the configuration file, gene panel file, gene-gene interaction (or reactome) data downloaded manually from Pathway Commons, cancer MeSH terms file, DrugBank information file, and the zip code file.

Figure 4 shows the system architecture of IDICAP in which three separate but dependent processes work together to generate cancer drug panel reports. The solid shaded components in Figure 4 represent the process of gathering drug information from DrugBank [17]. More details regarding the parsing of DrugBank information can be found in Section 4.2 below. The diagonally shaded components in Figure 4 represent the second process in which neoplasm-specific terms from Medical Subject Headings (MeSH) [16] are extracted. More details can be found in Section 4.3 below. The components inside the dashed box in Figure 4 comprise the crux of IDICAP, primarily integrating information pertaining to targeted cancer genes from ClinicalTrial.gov, DrugBank, and Pathway Commons’ PPI.

**Figure 5:**
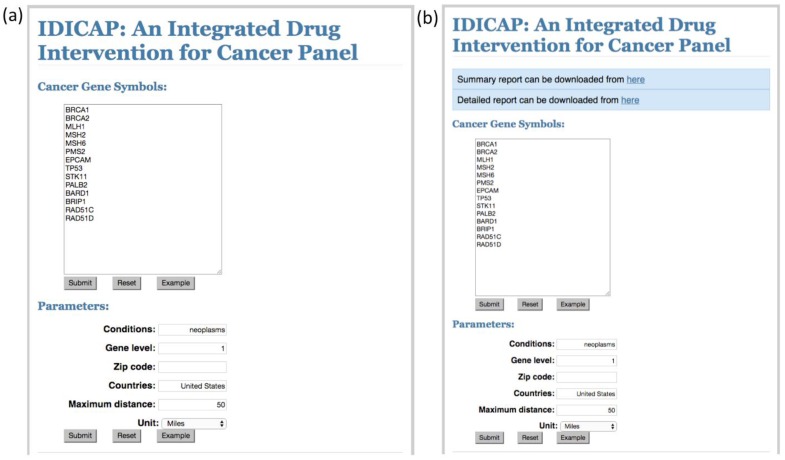
IDICAP web interface and control parameters. (**a**) User enters a list of human gene symbols in the **cancer gene symbols** box, gene symbols must be separated by at least a space, and they may span multiple lines. IDICAP will search for drugs and clinical trials that target these genes. **Conditions** box specifies the cancer type targeted by the clinical trials or drugs in neoplasm-specific MeSH terms. If the search involves more than one cancer type, separate the condition names by a comma (e.g., “breast cancer, ovarian cancer”). **Gene level** indicates the maximum level of protein-protein interaction to regress. See Section 4.6 for the details. **Zip code**, **countries**, **maximum distance**, and **unit** are parameters used by the proximity checking function. Refer to Section 4.5 for the detailed discussion. (**b**) Reports can be downloaded from the links displayed near the top of the web page.

Initially, IDICAP loads a handful of system parameters into the memory which control how the program processes the query. Among these system parameters, six parameters can be controlled by the user as shown in Figure 5a.

For each gene symbol, IDICAP will send a web request to the ClincialTrial.gov webserver with the parameters embedded in the URL as follows:

1. Search term is gene symbol,
2. The clinical trial is still open in recruiting participants,
3. Type of study is intervention,
4. The mean of intervention is drug, and
5. Disease condition is neoplasms or condition specified in the configuration file

The results returned from the website are XML files. These files are stored temporarily in the webserver for further processing. They will be purged automatically upon completion of the query. IDICAP parses the XML files for information relevant to the reporting requirements (see Section 4.4 for details). In the last step, IDICAP integrates clinical trial information with drug information through gene symbols and names of the drug undergoing clinical trial. Finally, two reports are generated based on the level of detail: a summary report and detailed report (Figure 2). Reports are formatted in tab-delimited format and they can be downloaded from the links displayed near to the top of the web page (Figure 5b). Users can reconstitute the readable format by importing it into popular spreadsheet tools, such as Microsoft Office or Google Sheets, by specifying tab as the delimiter. The IDICAP web service was developed using Python 2.7 and Flask 0.11 [25] under CentOS platforms.

### 4.2. DrugBank

As no real-time web query is provided by DrugBank, we downloaded drug information from the DrugBank website [26]. A Python program was developed to parse and extract drug information required for final reporting. Among the fount of information provided by DrugBank, IDICAP targets the fields tabulated in Table 3:

DrugBank holds over 8000 drug entries in its database including FDA approved drugs, nutraceuticals, and experimental drugs. As the goal of this project is targeted cancer therapy, our program uses the presence of neoplasm-specific MeSH terms in the pharmacology indication field of a DrugBank entry as a marker to determine whether or not a drug is a cancer drug. By using this condition, we have marked 494 drugs in DrugBank as cancer drugs. When IDICAP uses a gene to hunt for a drug that either targets the queried gene, its upstream regulator, or its counterpart in a complex, the drug is required to be tagged as a cancer drug by the method discussed above. IDICAP excludes nutraceuticals, over-the-counter products, drugs that have been withdrawn from the market, or drugs that are classified as illicit.

### 4.3. MeSH Terms

Medical Subject Headings (MeSH) is a thesaurus maintained by the National Library of Medicine (NLM). MeSH consists of a hierarchy of controlled vocabularies, namely MeSH terms, “used for indexing, cataloging and searching for biomedical and health-related information and documents.” [27]. The majority of entries in ClinicalTrials.gov are tagged with MeSH terms, indicating the conditions of the disease studied by the trial. For example, the trial NCT02491099 evaluates the activity of the drug afatinibin in patients with persistent or recurrent uterine serous carcinoma. Under the disease condition section, the trial is tagged by two MeSH terms: carcinoma, and cystadenocarcinoma. Note that the list of MeSH terms displayed in the ClinicalTrial.gov webpage may be different from the list if the output is an XML file.

MeSH terms are organized in a hierarchical structure with general terms above (roots) specific terms (leaves) in the hierarchy. For example, “neoplasms” is the most general term for all other cancer terms such as “Cysts”. A unique tree number “C04” is assigned to “neoplasms” in the MeSH hierarchy, meaning that tree number of all cancer-related MeSH terms will begin with the prefix “C04”. For example, the tree number of “Cysts” is “C04.182”, which begins with “C04”. For a special kind of cyst, e.g., “Bone Cysts”, its tree number consists of the prefix “C04.182” inherited from cysts and its unique reference “044”. The complete set of MeSH terms is available from the NLM Medical Subject Headings website [28]. We downloaded the 2016 MeSH Tree nodes with headings and scope notes [29]. A separate process (the diagonally shaded components in Figure 4) was designed to extract only neoplasm-specific MeSH terms, i.e., all MeSH terms where their tree numbers begin with “C04”, from the download file to another file. The full list of 671 cancer-related MeSH terms used by IDICAP can be found in Table S4 in Supplementary File.

### 4.4. Clinical Trials

IDICAP acquires the latest clinical trial information from Clinicaltrials.gov in real time as discussed above. Table 4 describes the fields of a clinical trial record that our tool examines:

### 4.5. Zip Codes

IDICAP can limit the location of clinical trials to be conducted in a single country or a list of countries. If the selected country is the United States, our tool accommodates users to further restrict clinical facilities to be located in the proximity (in miles or kilometers) of a zip code. These parameters (country, zip code, maximum distance, and unit) are user-specified (Figure 5a). The current version of IDICAP can only perform proximity checking for facilities in the United States. We will enhance our tool to handle proximity checking for other countries in future releases.

If the country of clinical facilities is limited to the United States and a non-zero value for the maximum distance parameter, IDICAP will calculate the distance between the location specified by the zip code and the location of the clinical facility using Haversine’s formulas stated below:

(1) $$hav\left(\frac{d}{r}\right)=hav\left(\theta_{2}-\theta_{1}\right)+\cos\left(\theta_{1}\right)\cos\left(\theta_{2}\right)hav\left(\lambda_{2}-\lambda_{1}\right)$$

(2) $$hav\left(\theta \right)=sin^{2}\left(\frac{\theta }{2}\right)=\frac{1-\cos\left(\theta \right)}{2}$$

(3) $$d=2r\;sin^{-1}(\sqrt{sin^{2}\left(\frac{\left(\theta_{2}-\theta_{1}\right)}{2}\right)+\cos\left(\theta_{1}\right)\cos\left(\theta_{2}\right)sin^{2}\left(\frac{\left(\lambda_{2}-\lambda_{1}\right)}{2}\right)}$$

- *d* is the distance between two points
- *r* is the radius of the earth i.e., 3959 miles or 6371 km
- *θ*_1_ and *θ*_2_ are latitude of points 1 and 2, in radians
- *λ*_1_ and *λ*_2_ are longitude of points 1 and 2, in radians

Geographic coordinates of zip codes were download from the US Census website [30]. For example, coordinates of zip code 18042 (Easton, PA, USA), and 19019 (Philadelphia, PA, USA) are (40.68, −75.22), (39.95, −75.16), respectively. Based on the calculation above, the distance between Easton and Philadelphia is approximately 50 miles or 80 km.

### 4.6. Reactome and NCI Pathway Interaction Database Information from Pathway Commons

Pathway Commons [21] is a hub of biological pathways aggregated from 25 data sources. Our protein-protein interaction data was obtained from two data sources through Pathway Commons: Reactome [31] and National Cancer Institute Pathway Interaction Database (NCI PID) [32]. Although there are 25 data sources, we chose only these two databases as they contain comprehensive protein-protein interactions (PPI) in human. However, if there are other PPI data sources that can improve our tool, we can readily incorporate them from Pathway Commons in the future. Broadly speaking, Reactome or PID is a collection of gene-gene or PPIs, i.e., the one-to-one relationship of how protein A interacts with protein B. Twelve types of interactions are annotated in the Reactome and PID as below:

1. Catalysis-precedes
2. Chemical-affects
3. Consumption-controlled-by
4. Controls-expression-of
5. Controls-phosphorylation-of
6. Controls-production-of
7. Controls-state-change-of
8. Controls-transport-of
9. Controls-transport-of-chemical
10. In-complex-with
11. Reacts-with
12. Used-to-produce

Reactome and NCI PID data were downloaded from Pathway Commons website [33]. Among many other biological pathway data files, our interest was focused on human Reactome and PID data stored in the files below:

- PathwayCommons.8.reactome.BINARY_SIF.hgnc.txt.sif
- PathwayCommons.8.pid.BINARY_SIF.hgnc.txt.sif

We developed a Python program to parse and merge the two PPI files listed above where gene symbol is the key. The output of the parsing process is the association of genes that interact with another gene. For instance, the Reactome data indicates that 143 genes interact with BRCA1 in the twelve ways stated above. If IDICAP cannot find a drug that targets a gene in DrugBank, it starts to search for genes that interact with the target gene and uses the interacting genes to search DrugBank again. By default, IDICAP regresses only one level. Users can alter the maximum level to regress by changing the parameter gene level (Figure 5a). Gene level with a value of 0 means no regressing. Gene level with a value of 2 means to regress two levels up from the queried cancer genes. Beware that computational time rises exponentially for each addition of gene level.

## 5. Conclusions

With the explosion of information, clinical studies, and treatments, physicians may find it challenging to prescribe an optimal combination of drugs that are tailored to the unique mutational profile of their patients. IDICAP is key in exploring new routes of disease treatment for cancer patients by thoughtful screening of information integrated from a myriad of reliable sources. Given the dynamic nature of clinical trial information, our tool delivers the most updated information to users through real-time queries. To the best of our knowledge, a similar service is unavailable for DrugBank, compromising the timeliness of drug information. Our plan is to implement this service in the future when it becomes available.

One of the major milestones of the Precision Medicine Initiative (PMI) is to capture an accurate genomic landscape of the diverse, healthy human by sequencing one million healthy people in the United States [34]. Genomic information obtained from this large-scale sequencing project will enable us to identify causal relationships between genetic variations and diseases. In the future, we will pursue the incorporation of high resolution variations of genes such as SNPs obtained from PMI and Catalogue Of Somatic Mutations In Cancer (COSMIC) [35] as search terms to identify relevant clinical trials and drugs that ameliorate targeted cancer therapy. We would also investigate the user experience design, namely UX, by seeking feedbacks from end users, thereby bringing this project closer to the ultimate goal of Precision Medicine Initiative.

## Figures and Tables

**Table 1:** Comparison of search results returned by IDICAP (Integrated Drug Intervention for CAncer Panel) and ClinicalTrials.gov. Columns ClinicalTrials.gov and IDICAP indicate the number of trials returned by the query of a gene with aforementioned search criteria.

Gene Panel	Genes	ClinicalTrials.gov	IDICAP	Remarks
Breast Cancer	*BRCA1*	19	16	Three trials were not returned by IDICAP for the following reasons: NCT02595905 is not recruiting, NCT01591746 is a breast reconstruction trial, and NCT01975363 is a dietary supplement trial.
	*BRCA2*	11	8	Same as above
	*TP53*	0	0	
	*PTEN*	6	5	All actively recruiting locations of NCT02162719 are outside the United States.
	*STK11*	0	0	
	*CDH1*	1	1	
	*PALB2*	1	1	
	*CHEK2*	1	1	
	*ATM*	2	2	
	*NBN*	1	1	
	*BARD1*	1	1	
Ovarian Cancer	*BRCA1*	9	7	NCT02477202 is device trial, and NCT02155777 has not yet begun recruiting patients
	*BRCA2*	5	3	Same as above
	*MLH1*	0	0	
	*MSH2*	0	0	
	*MSH6*	0	0	
	*PMS2*	0	0	
	*EPCAM*	0	0	
	*TP53*	2	2	
	*STK11*	0	0	
	*PALB2*	0	0	
	*BARD1*	0	0	
	*BRIP1*	0	0	
	*RAD51C*	0	0	
	*RAD51D*	0	0	

**Table 2:** Comparison of search results between DGIdb and IDICAP. Common results shared between the two tools are highlighted in bold.

Gene	DGIdb (DrugBank Only)	IDICAP
*ABL1*	1-[4-(PYRIDIN-4-YLOXY)PHENYL]-3-[3-(TRIFLUOROMETHYL)PHENYL]UREA	**BOSUTINIB**
	2-{[(6-OXO-1,6-DIHYDROPYRIDIN-3-YL)METHYL]AMINO}-N-[4-PROPYL-3-(TRIFLUOROMETHYL)PHENYL]BENZAMIDE	**DASATINIB**
	5-[3-(2-METHOXYPHENYL)-1H-PYRROLO[2,3-B]PYRIDIN-5-YL]-N,N-DIMETHYLPYRIDINE-3-CARBOXAMIDE	**IMATINIB**
	ADENOSINE TRIPHOSPHATE	**NILOTINIB**
	**BOSUTINIB**	**PONATINIB**
	**DASATINIB**	**REGORAFENIB**
	**IMATINIB**	XL228
	**NILOTINIB**	
	**PONATINIB**	
	**REGORAFENIB**	
*ATM*	CAFFEINE	CLADRIBINE
		FLAVOPIRIDOL
		XL844
*BRCA1*	-	CLADRIBINE
		FLAVOPIRIDOL
		AT7519
		XL844
		AT9283
		CYC116
		MLN8237
		BOSUTINIB

**Table 3:** List of fields in DrugBank targeted by our tool.

Field Name	Description	Example
DrugBank ID	Unique reference with prefix “DB”	DB00317
Molecule Type	Either “small molecule” or “biotech”	Small molecule
Description	General facts, composition and/or preparation of the drug molecule	
Name	Standard name of drug provided by the manufacturer	Gefitinib
Brand Names	Brand names from different manufacturers. Names are separated by a semicolon	Iressa;Tarceva
Prices	Unit cost drug prices in USD. Prices are separated by a semicolon	Tarceva 25 mg tablet USD 52.78; Iressa 250 mg tablet USD 68.08
Synonyms	List of alternate names separated by a semicolon	Iressa; gefitinib; ZD 1839
Pharmacology Indication	Identify neoplasm-specific MeSH terms.	Metastatic non-small lung cancer
Target Genes	Names of the gene targeted by the drug. Names are separated by a semicolon	*EGFR*
SNP Effects SNP Adverse	Known single nucleotide polymorphisms that may affect the potency of the drug. Multiple SNP effects are separated by a semicolon. A dash is shown if there is no known SNP effect.	G719A/C or ... ABCG2 ATP-binding ...

SNP: single nucleotide polymorphism.

**Table 4:** List of fields targeted by IDICAP.

Field Name	Description	Example
Trial ID	Unique reference with prefix “CT”	NCT02401347
Condition	Terms assigned to the disease	HER2/Neu Negative
Status	Must be recruiting	Open
Phase	Phase of the study	Phase 2
Drug Names	Name of drugs undergoing clinical trial	Talazoparib tosylate
MeSH Terms	MeSH terms tagged to the trial must appear in cancer-related MeSH term list	Breast neoplasms; triple negative breast neoplasms
Locations	Contact information of the facility conducting the clinical trial	Stanford University Hospitals and Clinics, Melinda L. Telli, M.D., Stanford, California, 94305, United States, peijenc@stanford.edu, 650-725-0866

## Supplementary Materials

The following are available online at www.mdpi.com/link, Table S1: Breast Cancer Panel, Table S2: Ovarian Cancer Panel, Table S3: Comprehensive Oncogene Cancer Panel, Table S4: Cancer MeSH terms, Table S5: Breast Cancer Drug Panel Summary, Table S6: Breast Cancer Drug Panel Report, Table S7: Ovarian Cancer Drug Panel Summary, Table S8: Ovarian Cancer Drug Panel Report, Table S9: Comprehensive Cancer Drug Panel Summary, Table S10: Comprehensive Cancer Drug Panel Report, Table S11: DGIdb vs IDICAP.
